# Dynamic risk allows us to adequately select patients with differentiated thyroid cancer who do not require radioiodine treatment

**DOI:** 10.20945/2359-3997000000374

**Published:** 2021-04-29

**Authors:** Erika Abelleira, Mirna Angela Peñaloza, Fernando Jerkovich, Fernanda Bueno, Fabián Pitoia

**Affiliations:** 1 University of Buenos Aires Division of Endocrinology Buenos Aires Argentina Division of Endocrinology, University of Buenos Aires, Buenos Aires, Argentina.

**Keywords:** Thyroid cancer, dynamic risk, without remnant ablation, structural incomplete response

## Abstract

**Objective:**

The treatment of patients with differentiated thyroid cancer (DTC) was modified in the last decade towards a more individualized approach according to the risk of recurrence (RR). We compared the outcomes of patients with low and intermediate RR (LRR and IRR) who received or did not receive radioiodine remnant ablation (RRA) after assessing the dynamic risk.

**Materials and methods:**

We included 307 DTC patients with LRR and IRR submitted to total thyroidectomy. All patients were reclassified according to the dynamic risk stratification (low or high). Patients with high dynamic risk received RRA (141 patients).

**Results:**

LRR patients who received RRA presented a frequency of structural incomplete response (SIR) of 5% at the end of the follow-up, compared to 2% in those who did not receive it (p=0.353). IRR patients treated with RRA had a frequency of SIR of 22%, compared to 5% in patients without RRA (p=0.008).

**Conclusions:**

This study demonstrates the usefulness of dynamic risk assessment to decide RRA in a cohort with a long-term follow-up. The lower prevalence of SIR at the end of the follow-up in patients who did not receive RRA highlights the adequate selection of those who would not benefit from RRA, even with an intermediate risk of recurrence.

## INTRODUCTION

Differentiated thyroid carcinoma (DTC) has an excellent prognosis, with 10-year overall survival above 95% (1). Total thyroidectomy (TT) followed by radioiodine remnant ablation (RRA) was the usual treatment for DTC (2). Currently, DTC therapy is decided according to the risk of recurrence (RR) of the disease (3–5). An individualized approach is recommended by the American Thyroid Association (ATA) guidelines (6) – among those of other societies (7–9) – in which postoperative assessing is suggested to determine the RR. These stratification systems consider the histopathological report, intraoperative findings, preoperative immediate and postoperative relevant data. The static classification of the initial RR could be improved by using the dynamic risk to decide RRA. The dynamic risk implies the re-stratification of the initial RR of DTC patients considering the different responses to the treatment – excellent, indeterminate (IR), biochemical incomplete (BIR), and structural incomplete (SIR) – using specific data obtained during the follow-up: thyroglobulin (Tg) and anti-thyroglobulin antibodies (TgAb) levels and results of imaging studies, including neck ultrasound (US), computed tomography (CT), and so forth, guided by the initial RR assessment. This strategy would provide a more accurate prediction of the RR and a more individualized approach (3).

Our hypothesis was that the correct selection of patients with a low dynamic risk in the first 12 months after surgical treatment would be associated with a lower frequency of structural incomplete response in the long-term follow-up, independently of the initial RR, and probably be associated with a higher prevalence of excellent response to treatment, so the aim of this study was to compare the outcomes of patients with an initial low and intermediate RR (LRR and IRR) who received or did not receive RRA after assessing the dynamic risk.

## MATERIALS AND METHODS

### Data source and study population

We retrospectively reviewed our database containing 551 files records of patients with DTC who were followed up on from January 2011 to June 2018 in the Division of Endocrinology, Hospital de Clínicas-University of of Buenos Aires after implementing the decision of remnant ablation based on the dynamic risk assessment. Inclusion criteria were the following: (i) age older than 18 years, (ii) adequate clinical and pathological data to allow an accurate determination of the initial RR, (iii) a low or intermediate RR, (iv) at least two measurements of thyroglobulin (Tg) and anti-thyroglobulin antibodies (TgAb) levels, and (v) a minimum follow-up of 12 months after initial treatment to enable defining the initial response to the therapy.

Of 419 patients with LRR and IRR, 50 were excluded because they were treated with lobectomy, 40 were excluded due to lack of follow-up (less than 12 months) and 22 were excluded due to insufficient data in the follow-up. With these criteria, 307 DTC patients were included in this study.

Each patient was stratified by using the eighth edition of the American Joint Committee on Cancer/International Union against Cancer (AJCC/UICC) staging system, and the risk of recurrence was assessed by using the modified risk stratification system from the 2009 ATA guidelines proposed by the American Thyroid Association (ATA) (low, intermediate or high) (6,10).

### Dynamic risk classification

**Figure 1:**
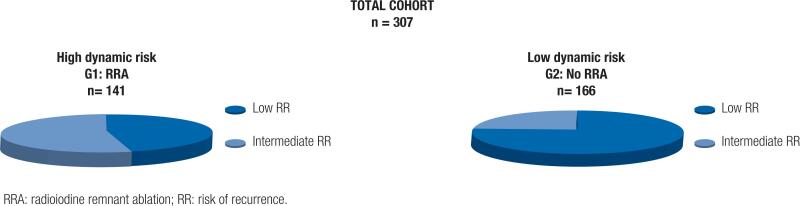
Dynamic risk classification.

After the initial response to treatment was determined, patients with initial LRR and IRR were reclassified into a low dynamic or high dynamic risk group according to the variables shown in Table 1 (3,5,11). They were divided into two groups – Group 1 (G1): n=141 patients who received RRA (patients with a high dynamic risk), and Group 2 (G2): n=166 patients who did not receive RRA (patients with a low dynamic risk) (Figure 1).

**Table 1:** Dynamic risk classification

Low dynamic risk	Initial excellent response
	Tg levels < 5 ng/mL under hormonal therapy without any suspicious ultrasonographic findings
	Indeterminate response with stable or declining TgAb levels
High dynamic risk	Initial structural incomplete response
	Indeterminate response with ultrasonographic suspicious findings
	Biochemical incomplete response with Tg > 5 ng/mL levels under hormonal therapy or increasing Tg levels during follow-up
	Biochemical incomplete response with increasing TgAb levels

Tg: thyroglobulin; TgAb: anti-thyroglobulin antibodies.

### Response to therapy assessment definitions based on initial therapy

The responses to therapy (initial response to treatment and the response at the end of the follow-up) were assessed according to the American Thyroid Association guidelines (6) and the classification proposed by Momesso and cols. (12) in G1 and G2, respectively.

### Clinical management during the follow-up

Each patient was assessed with Tg and TgAb measurements under hormonal therapy (at 30, 90, and 180 days after surgery) and a neck ultrasound every 6 months after initial treatment. Neck ultrasonography was performed using a 13-MHz linear transducer. Central and bilateral neck lymph node compartments and the superior mediastinum were inspected. Suspected lesions were evaluated by US-guided fine needle aspiration cytology (FNAC) and measurement of Tg and TgAb in washing fluid.

### Serum thyroglobulin and anti-thyroglobulin antibodies measurement

Serum Tg and TgAb were assessed using one commercial immunometric assay, and the same assay was used throughout a patient's follow-up. Serum Tg level was measured by Tg Electrochemiluminescence (EQLIA) Cobas e 411 (Roche) with analytical and functional sensitivities of 0.04 ng/mL and 0.1 ng/mL, respectively. A TgAb assay comprised the TgAb Electrochemiluminescence Cobas e411 (Roche). The serum TgAb level was considered negative when it was 20 IU/mL or lower, according to the manufacturer's recommendations.

### Ablation protocol

Our ablation protocol used fixed radioiodine activities based on the extent of the initial disease. Therapeutic doses of ^131^I ranged from 2.75 to 3.7 GBq (75-100 mCi ^131^I). A low-iodine diet was prescribed from one week before radioiodine administration through two days afterwards. Radioiodine was administered following that interval in all cases with thyroid hormonal withdrawal (THW) for at least 3 weeks, starting from thyroidectomy and TSH levels above 50 mIU/L. A post-therapy whole body scan (WBS) was performed 5–7 days after therapeutic radioiodine administration.

### Statistical analysis

Epidemiological data are presented as the mean ± standard deviation (SD) or as the median and range. Categorical variables are presented as percentages and absolute numbers. Categorical variables were compared using a chi-squared test or Fisher's exact test, and continuous variables using the Student's *t* test. The normal distribution of continuous data was confirmed using the Kolmogorov-Smirnov test. A p value < 0.05 was considered statistically significant. Hazard ratios and confidence intervals using log-rank analysis were also calculated. Univariate and multivariate Cox proportional hazards models were used with time to no evidence of disease as the outcome variable; the results were expressed as the hazard ratio (HR) with a 95% confidence interval. The initial risk was considered as a covariate. All statistical operations were performed using Stata 14.1 (Stata Corp, Texas, and USA).

## RESULTS

### Patient's characteristics

The demographic and clinical features of the included patients can be observed in Table 2. One hundred and forty-one patients received RRA after surgery, and one hundred and sixty-six patients did not. According to the ATA RR classification, those patients treated with RRA, 47% and 53% were considered to have low and intermediate RR, respectively, and among patients treated without RRA, these percentages were 75% and 25%.

**Table 2:** Baseline characteristics of 307 patients with differentiated thyroid cancer included in the study

Sex (%, n)		
	Female	83.4 (256)
	Male	16.6 (51)
Age (years)		
	Mean (SD)	46.1 (± 14.4)
Tumor size (cm)		
	Mean (SD)	1.4 (± 1.38)
Histology (%, n)		
	PTC classic	73 (224)
	PTC follicular	17 (53)
	PTC hidden sclerosing	2 (6)
	PTC oncocytic	3.3 (10)
	PTC tall cell <40%	2.7 (8)
	Follicular thyroid cancer	2 (6)
Primary tumor (T) (%, n)		
	T1a	39.4 (121)
	T1b	33.2 (102)
	T2	8 (25)
	T3a	6 (18)
	T3b	13.4 (41)
Regional Lymph Nodes (N) (%, n)		
	N0/Nx	72.6 (223)
	N1a	13.7 (42)
	N1b	13.7 (42)
AJCC/UICC Stage (%, n)		
	I	83.7 (257)
	II	16.3 (50)
Risk of recurrence (%, n)		
	Low	62.2 (191)
	Intermediate	37.8 (116)
Ablative radioiodine dose (mCi)		
	Mean (SD)	80.89 (± 17.86)
	Follow-up (months)	
	Mean ±SD	59.5 (± 22.31)

SD: standard deviation; PTC: papillary thyroid cancer; AJCC/UICC: American Joint Committee on Cancer/International Union against Cancer.

### Initial response to treatment and status at final follow-up in patients with initial low risk of recurrence with and without radioiodine remnant ablation (low and high dynamic risk, respectively)

The frequency of initial structural incomplete response (SIR) was 9% in patients treated with RRA and 0% in patients who did not receive RRA (p=0.002). The prevalence of SIR at the final follow-up was 5% and 2% in patients with and without RRA, respectively.

**Figure 2:**
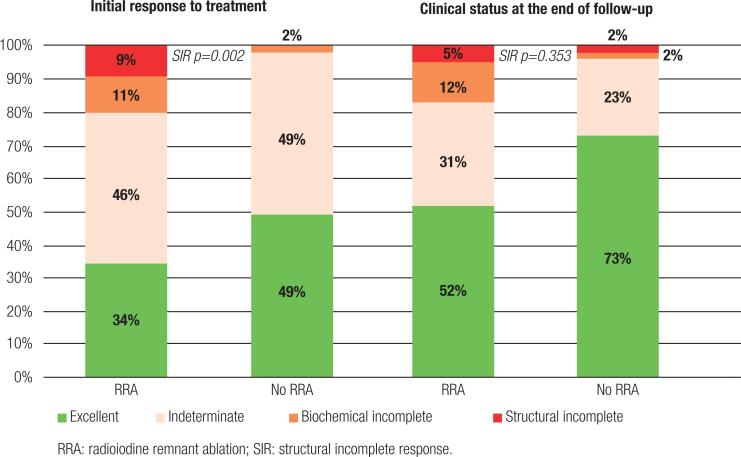
Response to therapy in patients with initial low risk of recurrence with and without radioiodine remnant ablation

Patients who received RRA had lower frequency of excellent response at the initial evaluation. The frequency of no evidence of disease (NED) at the end of the follow-up was 52% in patients treated with RRA and 73% in those who did not receive RRA (p=0.004) (Table 3 and Figure 2).

**Table 3:** Response to therapy in patients with initial low risk of recurrence with and without radioiodine remnant ablation

Patients with initial low RR	RRA (n = 67)	No RRA (n = 124)	P
**Initial response to treatment**			
Excellent response (%, n)	34 (23)	49 (61)	0.034
Indeterminate response (%, n)	46 (31)	49 (61)	0.408
Biochemical incomplete (%, n)	11 (7)	2 (2)	0.010
Structural incomplete	9 (6)	0 (0)	0.002
Clinical status at the end of follow-up			
NED (%, n)	52 (35)	73 (90)	0.004
Indeterminate (%, n)	31 (21)	23 (29)	0.154
Biochemical incomplete (%, n)	12 (8)	2 (2)	0.004
Structural incomplete (%, n)	5 (3)	2 (3)	0.353

RRA: radioiodine remnant ablation; NED: no evidence of disease.

### Initial response to treatment and status at final follow-up in patients with initial intermediate risk of recurrence with and without RRA (low and high dynamic risk, respectively)

The frequency of initial SIR was 24% in patients treated with RRA and 0% in patients who did not receive RRA (p=0.001). The percentage of SIR at the end of the follow-up was 22% in patients treated with RRA and 5% in those who did not receive RRA (p=0.008).

**Figure 3:**
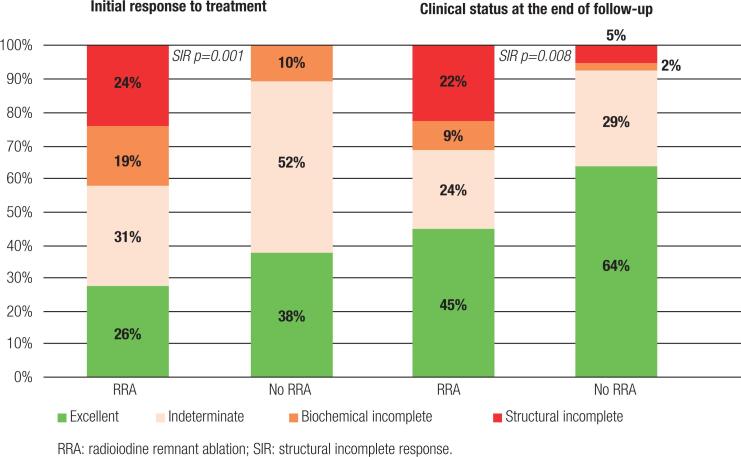
Response to therapy in patients with initial intermediate risk of recurrence with and without radioiodine remnant ablation

At the initial evaluation, the percentage of excellent response was similar in patients treated with and without RRA. Patients who received RRA had similar frequency of “no evidence of disease status” at the end of the follow-up to that among patients who did not receive RRA (Table 4 and Figure 3).

**Table 4:** Response to therapy in patients with initial intermediate risk of recurrence with and without radioiodine remnant ablation

Patients with initial intermediate RR	RRA (n = 74)	No RRA (n = 42)	P
**Initial response to treatment**			
Excellent response (%, n)	26 (19)	38 (16)	0.117
Indeterminate response (%, n)	31 (23)	52 (22)	0.020
Biochemical incomplete (%, n)	19 (14)	10 (4)	0.140
Structural incomplete	24 (18)	0 (0)	0.001
**Clinical status at the end of the follow-up**			
NED (%, n)	45 (33)	64 (27)	0.032
Indeterminate (%, n)	24 (18)	29 (12)	0.386
Biochemical incomplete (%, n)	9 (7)	2 (1)	0.143
Structural incomplete (%, n)	22 (16)	5 (2)	0.008

RRA: radioiodine remnant ablation; NED: no evidence of disease.

## DISCUSSION

The dynamic risk approach was initially proposed by Michael Tuttle (3). This stratification system constitutes a paradigm shift in the management of patients with DTC, mostly in those initially classified as harboring an intermediate RR or, less frequently, in patients with a high RR. In these patients treated with total thyroidectomy and RRA, an excellent response to therapy results in a significant decrease in the likelihood of having persistent or recurrent disease (from 18% to 2% in intermediate-risk and 66% to 14% in high-risk patients). Likewise, SIR is associated with an increased likelihood of having persistent structural disease or recurrence in each of the initial risk categories (3% to 13% in low RR, 18% to 41% in intermediate RR, and 66% to 79% in high RR) (3). Several observational and prospective studies validated the risk stratification system in patients treated with total thyroidectomy and RRA, showing similar results (5,13–22).

In 2014, Momesso and Tuttle (23) proposed the definition for the responses to treatment for DTC patients treated with lobectomy or total thyroidectomy without RRA. The first validation of the dynamic risk assessment in DTC patients without RRA was performed in 2016, showing similar results to those observed in patients who were treated with total thyroidectomy and RRA (12). Other studies reported a percentage of SIR between 1 and 2.9% at final follow-up in patients with low RR and intermediate RR who did not receive RRA (12,17,24–26).

Recently, we reported the responses to treatment in patients with low and intermediate RR in whom the decision for RRA was made immediately after surgery in comparison to the responses of patients who did not receive RRA due to the use of dynamic risk assessment (low dynamic risk). The frequency of SIR was 11.3% in patients treated with RRA and 0.9% in patients who did not receive remnant ablation, with a statistically significant difference (27). Our hypothesis regarding the high frequency of SIR in patients treated with RRA can be related to the percentage of subjects with a high probability of lymph node recurrence in the group treated with radioiodine that probably was not changed radically by the radioiodine administration.

In most patients treated without RRA included in a previous study, Tg levels evolved spontaneously to undetectable levels, demonstrating the benefit and usefulness of the dynamic assessment to decide RRA (27).

All the published studies addressing this topic are based on retrospective observational data; nevertheless, three prospective randomized, multicenter clinical trials that are currently ongoing will surely clarify this issue (28–30).

Our current study compared the outcome of DTC patients with low RR and intermediate RR who received RRA with the outcome of those who did not receive RRA in the dynamic risk. The frequency of SIR was higher in patients who did not receive RRA without a statistically significant difference when it was compared with those patients who received RRA. However, we found statistically significant differences in the dynamic risk when we compared the initial and final SIR in patients with initial low and intermediate RR who received RRA with those in patients who did not receive RRA. Five patients who did not receive RRA evolved with locoregional recurrence (cervical lymph nodes) during the follow-up, and all of them underwent a new cervical surgery. On the other hand, five patients with initial SIR who received RRA evolved without structural disease, but surgical treatment was necessary to achieve this status. Our hypothesis was that the RRA did not impact the frequency of SIR during the follow-up of patients who received RRA. One of the strengths of our study was the applicability of dynamic risk for RRA in a cohort with a long-term follow-up. The main limitation of our study was its retrospective design.

We observed that patients with intermediate RR who did not receive RRA considering the dynamic risk had a good prognosis, having a higher frequency of excellent response and lower prevalence of SIR at the end of the follow-up compared to those with initial intermediate RR. This clearly demonstrates that the dynamic risk stratification also allows for predicting the risk of structural incomplete response during the long-term follow-up in patients with initial intermediate RR who do not receive RRA.

In conclusion, this study demonstrates the usefulness of the dynamic risk assessment in the decision on radioiodine remnant ablation in a cohort with a long-term follow-up. The higher frequencies of excellent responses associated with a lower prevalence of structural incomplete response at the end of the follow-up in patients who did not receive remnant ablation highlights the adequate selection of those who would not benefit with this approach.
